# Protoenzymes: The Case of Hyperbranched Polymer-Scaffolded ZnS Nanocrystals

**DOI:** 10.3390/life10080150

**Published:** 2020-08-13

**Authors:** Irena Mamajanov, Melina Caudan, Tony Z. Jia

**Affiliations:** 1 Earth Life Science Institute, Tokyo Institute of Technology, Meguro, Tokyo 152-8550, Japan; melina.caudan@elsi.jp (M.C.); tzjia@elsi.jp (T.Z.J.); 2 Blue Marble Institute for Science, 1001 4th Ave, Suite 3201, Seattle, WA 98154, USA

**Keywords:** protoenzyme, hyperbranched polymers, photocatalytic nanoparticles, polymer-supported nanoparticles, metal-sulfide nanocrystals

## Abstract

Enzymes are biological catalysts that are comprised of small-molecule, metal, or cluster catalysts augmented by biopolymeric scaffolds. It is conceivable that early in chemical evolution, ancestral enzymes opted for simpler, easier to assemble scaffolds. Herein, we describe such possible protoenzymes: hyperbranched polymer-scaffolded metal-sulfide nanocrystals. Hyperbranched polyethyleneimine (HyPEI) and glycerol citrate polymer-supported ZnS nanocrystals (NCs) are formed in a simple process. Transmission electron microscopy (TEM) analyses of HyPEI-supported NCs reveal spherical particles with an average size of 10 nm that undergo only a modest aggregation over a 14-day incubation. The polymer-supported ZnS NCs are shown to possess a high photocatalytic activity in an eosin B photodegradation assay, making them an attractive model for the study of the origin of life under the “Zn world” theory dominated by a photocatalytic proto-metabolic redox reaction network. The catalyst, however, could be easily adapted to apply broadly to different protoenzymatic systems.

## 1. Introduction

Metabolism is one of the defining characteristics of life. The primary purpose of metabolism is the efficient utilization of external sources of energy to fuel cellular processes, such as growth and replication [1]. Enzymes, highly selective and efficient biocatalysts, govern metabolism. Enzymes offer a selective advantage to some reactions by enhancing their rate up to 10^23^-fold [2,3], unmatched by other types of catalysts, effectively shaping metabolic transformations. This level of control and order sets metabolism apart from any other set of chemical reactions. The crucial role of enzymes in life has prompted some to argue that catalytic enzyme precursors, or protoenzymes, played a critical role in the emergence of life [4,5].

Nearly all enzymes are based upon globular proteins, save for some RNA strands that have catalytic activity (ribozymes) [1]. While the discovery of the ribozyme [6,7] has become the basis of the RNA world origins of life theory [8,9], the question of how long, functional biopolymers were spontaneously formed remains unsolved. Previous studies have thus considered potentially more prebiotically reasonable transition metal complexes [10], small molecules [11], and mineral surfaces [12,13] as primitive protoenzymes. While metal cations and small molecules catalyze chemical reactions by lowering the activation energy or altering reaction pathways, macromolecular enzymes offer an additional advantage of selective binding, orienting, and enclosing reactants within a modulated microenvironment [14]. In the prebiotic world, in the absence of long coded proteins or RNA, mineral surface catalysis could have performed some catalytic functions. Encapsulation of organic molecules between mica sheets has been proposed to trigger protoenzymatic processes [15], and clay minerals have been shown to facilitate the polymerization of activated nucleotides, presumably by orienting the reactants [16]. The orientation of glycine molecules on oxide mineral surfaces has also been shown to affect its reactivity towards polymerization [17]. In the realm of organic biomimetic catalysts, both prebiotic and synthetic, the utility of amino acids and small peptide aggregates has been extensively explored (Reference [18], and references therein). In the prebiotic context, Gazit and coworkers have shown both that aromatic dipeptide assemblies stabilize RNA [19] and that assemblies of phenylalanine and zinc cations exhibit strong hydrolase activity [20]. Mansy and coworkers have investigated the redox activity of short peptide and iron-sulfur cluster nanosized assemblies as a putative origin of ferredoxins [21,22].

Prebiotic reaction systems are known to produce messy intractable mixtures of macromolecular material, or "tar" [23]. Sidney Fox has pioneered the concept of messy protoenzymes based on the tarry material his "proteinoid world" hypothesis that concentrated on the microsphere structures (proteinoids) formed upon thermal condensation of amino acids. Fox and coworkers had shown that dry-heated mixtures of amino acids rich in glutamic acid yield likely cross-linked or network polymeric materials which resulted in proteinoid assembly [24,25]. Such proteinoids have also been found to possess marginal catalytic activities towards hydrolysis reactions [26,27,28]. Ryan and Fox then showed that a combination of proteinoids and Mg^2+^ cations had a slightly higher catalytic activity towards the activation of glycine by ATP than Mg^2+^ cations alone [29]. In follow-up work, Quirk had shown that the addition of divalent cations augmented the capability of proteinoids to catalyze the hydrolysis of phosphodiester bonds [30]. 

To further study the catalytic capacity of tarry polymers, herein we explore one possible subset of tarry polymers, highly branched or hyperbranched polymers, as potential protoenzymes. Hyperbranched polymers are a type of dendritic polymer characterized by a high branching density with the potential of branching in each repeating unit [31]. These polymers are characterized by high solubility and low viscosity compared to their linear counterparts [31]. It is worth noting that other types of branched polymers, distinct from hyperbranched polymers, also exist and might conceivably be present in tarry polymers. These other branched polymers include mostly linear polymers with few branching imperfections with properties close to those of linear polymers, as well as mostly insoluble, low glass transition cross-linked and network polymers, structures containing a low and a high number of linkages between polymer chains, respectively [32]. Here, we concentrate specifically on hyperbranched polymers because they have been long considered as biomimetic catalysts in synthetic applications [33]. In particular, the globular structure of hyperbranched polymers is conducive to creating modulated microenvironments, and the multitude of end groups in these structures also allows for efficient substrate binding [34]. In synthetic laboratories, hyperbranched polymers are usually prepared in a one-pot synthesis, with limited control over the molar mass and branching accuracy, and lead to "heterogeneous" products with distribution in molar mass and branching [34]. Our previous studies have demonstrated the plausibility of the prebiotic synthesis of hyperbranched polyesters from citric acid and glycerol [35,36,37] and the ability of hyperbranched polyesters to catalyze the Kemp elimination reaction [38].

In the synthetic community, the application of enzymatic systems to modern synthetic processes has become a desirable goal [39]. The high cost of enzyme isolation and enzyme incompatibility with synthesis conditions outside of physiological has prompted studies of non-biopolymer-based enzyme mimics. Approaches to the design of artificial enzymes range from stripping the cofactors of the biopolymeric scaffold, synthesis of supramolecular active sites, surveying enzyme-mimetic properties of various nanoparticles, and investigation of non-biological polymeric scaffolds [40]. In the context of prebiotic chemistry, it is conceivable that the first enzymes may not have been purely or entirely proteinaceous or RNA-based. Herein, we describe an effort to combine the principles of artificial enzyme construction discovered by synthetic chemists to gain new insights into the question of the provenance of enzymes.

Catalytic nanoparticles are an attractive avenue to explore in the context of protoenzymes. The catalytic properties of nanoparticles, in particular, arise from their large surface area compared to the nanoparticle’s total number of atoms [41]. Many nanoparticle catalysts have been shown to catalyze enzymatic reactions to earn the term "nanozyme" [42]. Nanomaterials are usually thought of as synthetic or anthropogenic; however, they can also form through natural abiotic processes and could have been present on prebiotic Earth. All mineral formation processes undergo a sometimes-persistent nanophase stage during formation. Volcanic ash clouds contain polydisperse particles that range from 100 to 200 nm in size and are primarily composed of silicate and iron compounds [43]. In geological processes, nanoparticles can be generated through mineral weathering or by mechanical grinding associated with earthquake-generating faults in the Earth’s crust [44]. We have investigated the potential role of metal-sulfide nanocrystals (NCs) as possible precursors to metal sulfide clusters found in some modern enzymes. Metal sulfide deposits near deep-sea hydrothermal vents have become the basis of the iron-sulfur world theory [45]. The model suggests that these minerals would catalyze complex sequences of reactions, driven by the energy from the vents, eventually leading to life. In the subaerial "Zinc world" scenario [46], life emergence would have been driven by light energy harnessed by photoactive semi-conductive zinc sulfide (ZnS) minerals. Under "Zinc world" assumptions, the geochemical formation of long-lived ZnS nanoparticles would extend the catalytic capacity of the material due to the significantly increased specific surface area of the catalyst and the exciton confinement effects [47]. NCs, however, tend to spontaneously aggregate; therefore, it is a common practice to include polymeric supports [48] or other capping agents [49,50] in NC formulations to decrease particle overgrowth and aggregation. Here, we report on the formation of photocatalytic hyperbranched polymer-scaffolded ZnS NCs.

## 2. Materials and Methods 

All chemicals were purchased from Sigma-Aldrich (St. Louis, MO, USA) and used without further purification.

### 2.1. Synthesis of Hyperbranched Polyethyleneimine (HyPEI)-Supported ZnS Nanocrystals (NCs)

**Scheme 1:**
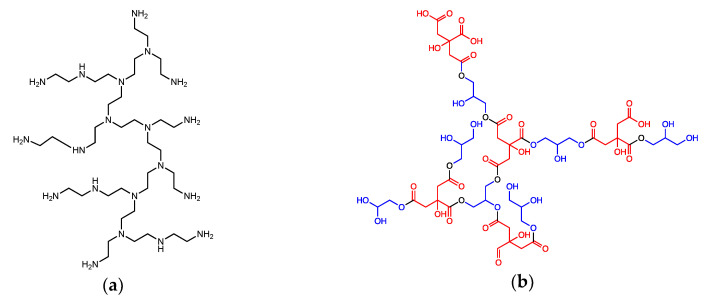
The structure of representative isomers of (**a**) hyperbranched polyethyleneimine (HyPEI, M_w_ = 834 Da), and (**b**) hyperbranched glycerol (G) citrate (C) polyester (G_7_C_5_, M_w_ = 1406 Da). Glycerol and citrate moieties are colored in blue and red, respectively.

The HyPEI-supported ZnS NCs were synthesized immediately prior to analyses or catalytic assays. The procedure for the preparation of hyperbranched polyethyleneimine (HyPEI, Scheme 1a) was adapted from a protocol described by Hassan and Ali [51]. The protocol devised by Hassan and Ali utilized high molecular weight HyPEI (average M_w_ ~25,000–50,000); however, we used HyPEI of a low molecular weight HyPEI (average M_w_ ~800 Da, Sigma-Aldrich 408719) comparable to that of hyperbranched polyesters synthesized under drying conditions [35,36]. Briefly, to produce 10 mg of supported ZnS NCs, a solution of 0.4 g HyPEI in 124.9 mL water (pH = 6) was titrated with 2.56 mL of 40 mM ZnCl_2_ solution and magnetically stirred for 1 h. 2.56 mL of a 40 mmol solution of Na_2_S was then added dropwise and the resulting mixture was allowed to stir magnetically for 1 h.

### 2.2. Synthesis of Glycerol Citrate Polyesters

A polyesterification to form glycerol citrate polyesters (Scheme 1b) was conducted, starting with 10 mL of an aqueous solution containing 330 mM citric acid and 660 mM glycerol. Some formulations also contained 165 mM CoCl_2_ or ZnCl_2_. The samples were incubated, uncovered, at 85 °C for 72 h. The resulting gel-like product was re-dissolved in 10 mL of water and dialyzed against deionized water through a 500–1000 Da cut-off membrane using a 10 mL-capacity Float-A-Lyzer device (Repligen, Waltham, MA, USA) over 24 h. The water was exchanged twice, after 2 and 16 h, respectively. The resulting solution was lyophilized in an Eyela FDU-1200 freeze-dryer (Bunkyo-ku, Tokyo, Japan) overnight. The dry product was stored in a sealed container at 4 °C until further use. The detailed spectroscopic, chromatographic, and mass-spectrometric characterization of these polymers is described elsewhere [34].

### 2.3. Measurement of the Divalent Cation Loading Capacity of the Polymers

100 mg of glycerol citrate polyester (formulation without added cations), COOH-terminated oligo-2,2-bis(hydroxymethyl)propionic acid dendrimer; trimethylol propane core, generation 1, C_45_H_62_O_30_ (Sigma-Aldrich, 806099), OH-terminated oligo-2,2-bis(hydroxymethyl)propionic acid dendrimer; trimethylol propane core, generation 1, C_21_H_38_O_12_ (Sigma-Aldrich, 805920) polyester dendrimer; and HyPEI was equilibrated overnight in 1 mL of a 165 mM aqueous solution of CoCl_2_. This step was omitted for the glycerol citrate polyesters prepared in the presence of CoCl_2_. The solutions were then dialyzed against deionized water through a 500–1000 Da cut-off membrane using a 1 mL-capacity Float-A-Lyzer device over 24 h. The water was exchanged twice, after 2 and 16 h, respectively. The resulting solutions were lyophilized as described above. The content of Co^2+^ per unit weight of the resulting polymer was determined spectroscopically. Spectra of six CoCl_2_ aqueous solutions at pH = 2.5 (to match the pH of glycerol citrate polyester solutions) at concentrations ranging from 1 to 40 mM were measured at 25 °C utilizing a JASCO (Hachioji, Tokyo, Japan) V-670 UV-vis spectrometer. The absorbance values at λ_max_ of 511 mm were plotted against the CoCl_2_ concentrations and linearly fitted to derive the extinction coefficient of 5.6 × 10^−2^ Mm^−1^ cm^−1^ in accordance with the Beer–Lambert law. 50 mg samples of Co^2+^-bearing glycerol citrate, HyPEI, and dendrimer solutions were dissolved in 1 mL water and adjusted to pH = 2.5 with concentrated HCl (if required). The absorbance values at λ_max_ of the resulting solution, also at 511 nm, were substituted into the Beer–Lambert equation to derive the Co^2+^ concentrations in the samples. The values normalized to polymer weight are reported below.

### 2.4. Synthesis of Glycerol Citrate Polyester-Supported ZnS NCs

The loading capacity towards the absorption of Zn^2+^ was estimated to be the same, or similar, as in the case of Co^2+^ ions. An excess of the theoretical amount of the Zn^2+^-bearing polyester was used. 0.8 g of Zn^2+^-bearing polyester (instead of the theoretical value of 0.56 g) was dissolved in 27.4 mL of water. The pH of the solution was adjusted to pH = 6 with NaOH. 2.56 mL of a 40 mmol solution of Na_2_S was then added dropwise and was allowed to stir for 1 h magnetically.

### 2.5. Mass Spectrometry

Matrix-assisted laser desorption/ionization mass spectrometry (MALDI-MS) spectra were collected on an ultrafleXtreme Bruker Daltonics MALDI-time-of-flight (MALDI-TOF)-MS (Bruker Corporation, Billerica, MA, USA) in positive ion mode. External mass calibration was conducted using proprietary peptide mixtures provided by Bruker Corp. The sample preparation matrix, a 90:10 mixture of 2,5-dihydroxybenzoic acid and 2-hydroxy-5-methoxy benzoic acid (SDHB), was dissolved in deionized water. Subsequently, the lyophilized polymeric samples and the matrix were mixed at a 1:10 (v/v) ratio in advance, and then the mixture was applied to the plate before analysis.

### 2.6. Transmission Electron Microscopy (TEM)

Transmission electron microscopy and field-emission (FE)-TEM imaging were performed at the Tokyo Institute of Technology Materials Analysis Division Technical Department in Meguro-ku, Tokyo. Samples were prepared by placing a droplet of ZnS nanoparticle solution (see above for synthesis method) on a Collodion Film COL-C15 copper grid (Okenshoji Co., Ltd., Chuo-ku, Tokyo, Japan) and wicking away the liquid to deposit a thin layer onto the grid. The grid was allowed to dry at room temperature for 2 days before imaging. After confirmation using a Hitachi (Chiyoda-ku, Tokyo, Japan) H7650 Zero A TEM (100 kV), high-resolution imaging was performed at room temperature on a JEOL (Akishima, Tokyo, Japan) JEM-2010F FE-TEM at 200 kV with the following specifications: a ZrO/W(100) Schottky cathode, ultra-resolution pole piece, Gatan (Sarasota, Florida, USA) Digiscan System Model 688, Gatan MSC 794 CCD Camera, and an EDAX (Minato-ku, Tokyo, Japan) Genesis energy dispersive X-ray spectrometer.

### 2.7. Photocatalytic Activity Measurement

**Scheme 2:**
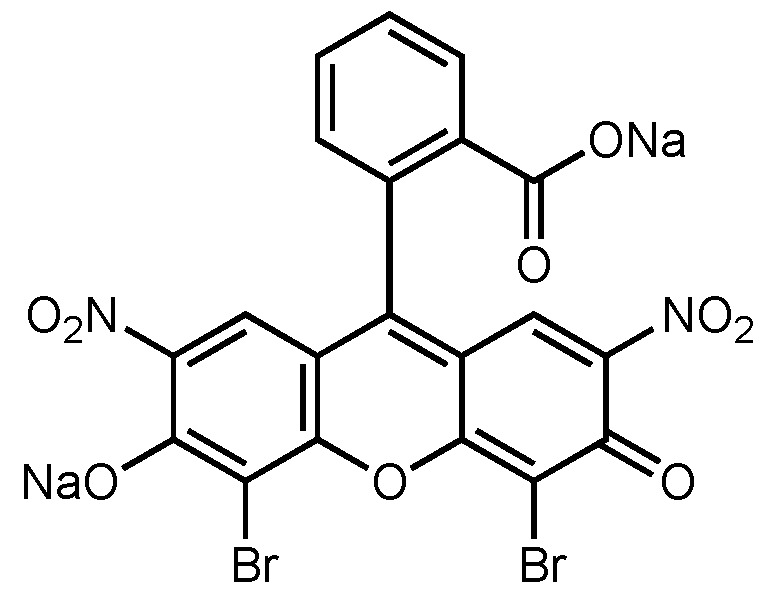
The molecular structure of eosin B. The photodegradation is assumed to proceed via a homolysis of an excited dye into radicals followed by radical degradation [53].

The procedure was adapted from Hu et al. [52]. A jacketed Pyrex flask (capacity ca. 40 mL) was used as the photoreactor vessel. For a typical experiment, a solution adjusted to pH = 6 by NaOH containing eosin B (Scheme 2, 5.0 × 10^−5^ M, 30 mL) and polymer-supported ZnS NC catalysts (10 mg of ZnS, corresponding amount of polymer) was magnetically stirred in the dark for 30 min to reach the adsorption equilibrium of eosin B with the catalyst and then exposed to light from a 100 W high-pressure mercury lamp (Handy 100, Mizuka Planning, Amagasaki, Hyogo, Japan). The temperature of the solution was maintained at 4–5 °C by circulating ice water through the flask’s jacket. A 200 μL aliquot was collected every 15 min. UV/Vis absorption spectra of each aliquot were recorded on a JASCO V-670 UV-vis spectrometer.

## 3. Results

**Figure 1:**
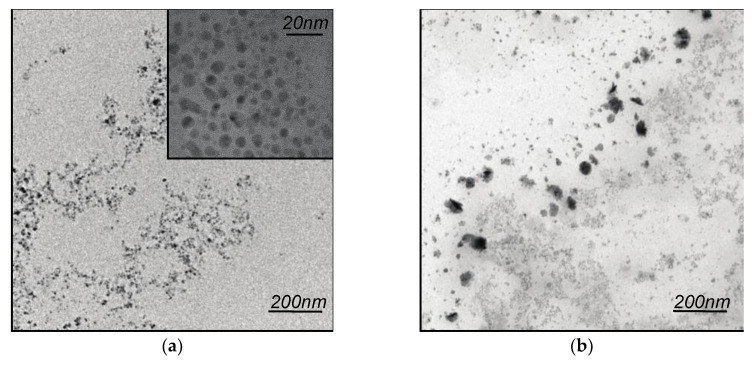
Transmission emission microscope (TEM) images of HyPEI-supported ZnS nanocrystals. (**a**) Low-magnification image of a freshly prepared sample. Inset: high-magnification field-emission (FE)-TEM micrograph of a freshly prepared sample. (**b**) Low-magnification image of a sample aged for 14 days. Additional TEM views of the above samples are shown in Supplementary Figure S1.

In the HyPEI-supported ZnS NC preparation experiments, when Na_2_S solution was titrated into a solution of ZnCl_2_ at neutral pH, the clear solution quickly turned cloudy with a white precipitate, presumably crystalline zinc sulfide (ZnS), settling within an hour. However, when Na_2_S solution was titrated into a ZnCl_2_ solution in the presence of hyperbranched HyPEI (at neutral pH), the resulting solution remained unclouded for at least 14 days, suggesting that the ZnS product remained soluble. Figure 1a shows a low-magnification TEM (transmission emission microscope) micrograph of a freshly prepared ZnS/HyPEI sample. The high-magnification FE (field-emission)-TEM micrograph in Figure 1a (inset) reveals spherical particles with an average size under 10 nm. The low-magnification TEM micrograph in Figure 1b shows the presence of both ~10 and ~50 nm aggregated particles in a solution of ZnS/HyPEI that was kept at room temperature for 14 days. The ZnS/HyPEI particulates remain despite some aggregation also existing in the nanometer range.

Next, we considered hyperbranched polyesters formed upon the condensation between citric acid and glycerol, as well as with commercially available oligo-2,2-bis(hydroxymethyl)propionic acid (bis-MPA) dendrimers terminated with either carboxylic acid or alcohol functional groups. When we attempted the same procedure for the preparation of scaffolded ZnS NCs, a large amount of white precipitate formed immediately upon the addition of Na_2_S solution. Neither commercial polyester dendrimers nor the glycerol citrate polymer exhibited high affinity towards transition metal binding (Table 1). The citric acid glycerol polymers formed in the presence of CoCl_2_ retained a significant amount of Co^2+^ through dialysis.

The glycerol citrate polymer prepared in the presence of Zn^2+^ was used for further experiments. The mass spectral analysis of this polymer mixture (Supplementary Figure S2) revealed a heterogeneous mixture of polymeric products up to ~1400 Da. The resulting polymer solution was titrated with Na_2_S to form polymer-scaffolded ZnS NCs.

**Figure 2:**
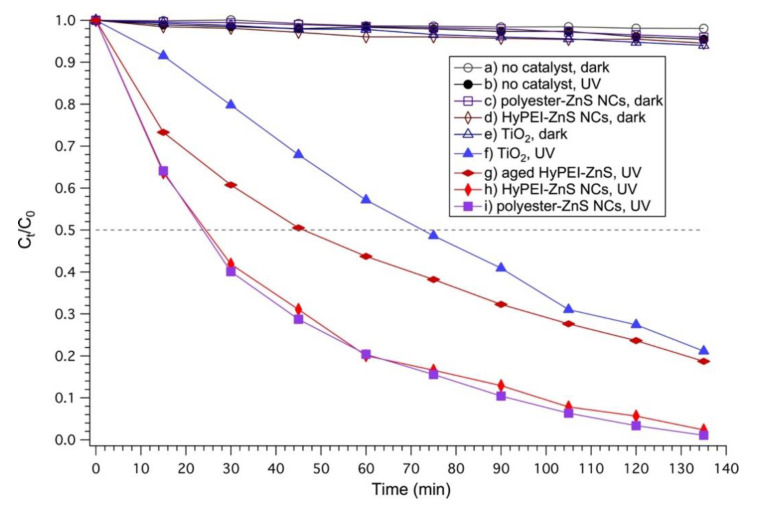
Time-lapse measurement of photodegradation of eosin B (5.0 × 10^−5^ M, 30 mL) under different conditions: (a) without a catalyst, in the dark, (b) without a catalyst, under UV irradiation, (c) with polyester-supported ZnS NCs (10 mg of NCs), in the dark, (d) with HyPEI-supported ZnS NCs, in the dark, (e) with 21 nm TiO_2_ nanoparticles, in the dark, (f) with 21 nm TiO_2_ nanoparticles, under UV, (g) with HyPEI-supported ZnS NCs, aged at room temperature for 14 days, under UV, (h) with freshly prepared HyPEI-supported ZnS NCs, under UV, and (i) with polyester-supported ZnS NCs, under UV. When appropriate, 10 mg of TiO_2_ nanoparticles or ZnS NCs was used. The normalized concentration (C_t_/C_0_) was derived from the UV absorbance values at 520 nm.

To demonstrate the potential applicability of the polymer-supported ZnS NCs to the prebiotic chemistry in “Zinc world” settings, we have followed the analytical procedure described by Hu et al. [52] used to explore the photocatalytic activity of ZnS NCs and nanoporous nanoparticles without an organic support. We investigated the photocatalytic activity of the polymer-supported ZnS NCs relative to those of bulk ZnS and the commercial photocatalyst TiO_2_ (21 nm), with the photocatalytic degradation of eosin B as a test reaction. We have chosen the characteristic absorption of eosin B at 520 nm as the monitored parameter for the photocatalytic degradation process. 30 mL of a 5.0 × 10^−5^ M solution of eosin B was subjected to incubation with periodic sampling under the following conditions (Figure 2, Supplementary Figure S3): (a) without a catalyst, in the dark, (b) without a catalyst, under UV irradiation, (c) with polyester-supported ZnS NCs (10 mg of NCs), in the dark, (d) with HyPEI-supported ZnS NCs, in the dark, (e) with 21 nm TiO_2_ nanoparticles, in the dark, (f) with 21 nm TiO_2_ nanoparticles, under UV, (g) with HyPEI-supported ZnS NCs, aged at room temperature for 14 days, under UV, (h) with freshly prepared HyPEI-supported ZnS NCs, under UV, (i) with polyester-supported ZnS NCs, under UV, (j) with unsupported ZnS_2_, and (k) with monomeric citric acid, glycerol, ZnCl_2_, and Na_2_S, under UV. When appropriate, 10 mg of TiO_2_ nanoparticles or ZnS NCs was used. Under the experimental conditions from (a) to (e), the photocatalytic effect on the solution degradation without catalysts but under exposure to UV light was almost the same as that with catalyst but no exposure to UV light. For example, a slight decrease in the concentration of eosin B was detected in the absence of any catalyst (Figure 2, curve b). Exposure to UV light for 135 min resulted in only 5–6% degradation of the dye with and without the catalyst. The curves measured under the experimental conditions (f) to (i), those containing a catalyst and with exposure to UV light, are indicative of a significantly higher catalytic activity. For example, the fresh HyPEI-supported ZnS NC (Figure 2, curve h) sample was completely decolorized after 135 min of exposure to UV light with a half-life of ~24 min. The aged HyPEI-supported ZnS NC (Figure 2, curve g) and commercial TiO_2_ particle (Figure 2, curve f) samples exhibit a lower photocatalytic activity with half-lives of ~45 and 72 min, respectively. This difference in the photocatalytic activity between the fresh HyPEI-ZnS NC sample and aged HyPEI ZnS NC and TiO_2_ samples can be explained by the smaller diameter of the ZnS NC (~10 nm of the fresh HyPEI-ZnS NCs versus 20 nm of TiO_2_ particles versus ~50 nm of the aged HyPEI-ZnS NCs), hence resulting in a larger catalytic surface area. The polyester-supported ZnS NCs activity (Figure 2, curve i) closely resembles that of the fresh HyPEI-supported ZnS NCs (Figure 2, curve h). Unsupported ZnS undergoes quick aggregation and precipitation under the irradiation (Supplementary Figures S3 and S4). In this sample (Supplementary Figure S3, curve j), the solution was rapidly decolorized. However, the dye was adsorbed and preserved on the surface of the precipitated ZnS particles, suggesting that decolorization was derived from the solid–liquid phase partitioning rather than photodegradation (Supplementary Figure S3). The photocatalytic activity of polyester-supported ZnS NCs was further compared to that of a solution containing unreacted citric acid and glycerol, as well as ZnCl_2_ and Na_2_S (Supplementary Figure S3, curve k). ZnS did not precipitate out of the solution containing citric acid and glycerol as it did in a neat solution. Moreover, the citric acid, glycerol, ZnCl_2_, and Na_2_S solution did not exhibit a detectable photocatalytic activity. It is conceivable that the process of chelation of zinc cations by the unreacted citric acid in the sample (i) interferes with the formation of ZnS particulates.

## 4. Discussion

**Scheme 3:**
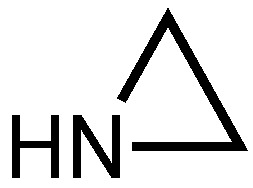
The molecular structure of aziridine.

Our results show that hyperbranched PEI- and glycerol citrate polyester-supported ZnS NCs make excellent photocatalysts. Their straightforward formation makes them plausible catalytic agents before the onset of protein-based biocatalysis. HyPEI contains abundant primary, secondary, and tertiary amine groups, and has a strong affinity towards the complexation of transition metals [54]. Consequently, HyPEI has been used in water treatment and as a support for nanoparticles [51,55]. In synthetic laboratories, HyPEI is prepared by ring-opening polymerization of aziridine (Scheme 3) in 1M aqueous HCl at 20–80 °C [56]. The synthesis catalyzed by Lewis acids has been reported to proceed in aqueous solution at 90–110 °C [57]. The starting material, aziridine (Scheme 3), and its derivatives have been previously discussed as potentially prebiotic material as it has been tentatively detected in the interstellar space [58]. Aziridine has also been proposed to be an intermediate in a model prebiotic synthesis of l-histidine [59]. The direct prebiotic synthesis of HyPEI, however, is yet to be investigated. As for the prebiotic synthesis of hyperbranched glycerol citrate polyesters, we have previously shown their formation in drying reactions at 85 °C [35] and under wet–dry cycling conditions [36,37]. In this study, we prepared the glycerol citrate sample following the drying procedure described previously [35]. The products of the previous study were a mixture of species ranging from tetramers to dodecamers, as evidenced by high-resolution mass spectra [35]. The previous study contained polyester samples prepared neat or in the presence of various divalent cations. In all cases, according to quantitative ^1^H and ^13^C NMR (Nuclear Magnetic Resonance), the majority of carboxylic acid groups were esterified, suggesting that the majority of citric acid moieties became a focal point for branching [35]. The formulations containing the cations showed a slightly higher number of unreacted carboxylic groups [35]. No evidence of significant degradation citric acid, glycerol, or polymeric products had been detected [35]. The presence of divalent cations during citric acid and glycerol polyesterification shows the effect of templating by altering the structure and the size of the resulting polymer [35]. The cation-containing formulations resulted in shorter polymers that incorporated more citric acid moieties compared to neat solution formulations. Stochiometrically, the increased number of citric acid moieties, in turn, increases the number of unreacted carboxylic groups changing the metal affinity. We note that the above analysis is based on chemical structure information of glycerol citrate gleaned from previous studies [35]. The glycerol citrate used in this study, while synthesized under the same conditions, has not been subjected to the same array of analyses, and some dissimilarities are possible. Nevertheless, the mass spectrometry data collected using the present batch of polyesters (Supplementary Figure S2) is consistent with the previous report, suggesting that the glycerol citrate synthesized within this study at least approximates the chemical structure of the previously analyzed glycerol citrate. The synthesis of the metal templated polyesters before the synthesis of the NCs required additional steps, potentially making this polyester system less prebiotic. It must be pointed out, however, that in this study, we have set out to investigate the utility of hyperbranched polymer-scaffolded catalysts as a model system in prebiotic chemistry, and their synthesis and behavior in a laboratory simulation mimicking prebiotic conditions has not been studied thus far.

Both the glycerol citrate and HyPEI discussed in these studies fall within the range of oligomers or short polymers. The formation of low molecular weight species is consistent with many conditions discussed in the context of prebiotic chemistry (e.g., References [60,61]). The identification of functional polymers within the low molecular weight range (rather than a higher molecular weight range) is, therefore, significant to origins of life research. In fact, hyperbranched polymers tend to undergo gelation with increasing molecular weight, forming extensive networks between strands through cross-linking [62]. The gelation is associated with a drastic change in polymer properties, such as decreased solubility and increased viscosity [31], potentially resulting in inhibition of enzyme-like catalytic function, and would also position the product within the network polymer classification rather than the hyperbranched polymer classification. Polymerization of AB_x_-type monomers, where A and B denote mutually reactive functional groups, can theoretically proceed infinitely without the occurrence of gelation based on Flory’s statistical theory of mass distributions of three-dimensional polymers [62]. This prediction appears to hold for the HyPEI, at least for molecular weights of up to 50,000 Da [51]. The glycerol citrate, which is not a product of polymerization of AB_x_-type monomers, does undergo gelation upon prolonged heating [36]. The onset of gelation, and potential inhibition of catalytic function, of glycerol citrate can be delayed under wet–dry cycling [36].

Since neither hyperbranched nor other branched polymers resemble the structure of any of the major biopolymers, the origin of life community has generally considered them to be undesirable products [63], and as such, their function has not been explicitly studied in the context of prebiotic chemistry. A case in point is the heavily criticized [64], in part for producing non-linear polymers, work on proteinoids conducted by Fox. Fox’s studies focused on dry-heated mixtures of amino acids rich in glutamic acid [24,25]. Fox claimed that proteinoids consisted of linear polypeptides [65], despite the presence of multifunctional monomers. However, the proteinoids were never sufficiently analyzed to definitively make this claim. Nevertheless, since a multifunctional amino acid, aspartic acid, does not form a branched product upon thermal polymerization [66], the claim of proteinoid linearity might not have been unreasonable. It is also possible that proteinoids contained a fraction of either sparsely or highly branched polymers, or both. Given the high molecular weight [24,25] and the apparent poor solubility of proteinoids, they are likely to have consisted of a combination of predominantly linear strands, cross-linked polymers formed through linking the linear or sparsely branched chains, and network polymers formed upon the gelation of branched polymers, but not hyperbranched polymers. Furthermore, since amino acids in Fox’s experiments were subjected to heating at 160–190 °C [24,25], it is possible that both monomers and polymeric products had undergone degradation 

While we so far have only explored the catalysts and the processes relevant to the "Zinc World" scenario, the demonstrated synthesis of the photocatalytic complexes could be applicable in a variety of geological settings. Therefore, by considering different particles or cofactors, as well as different plausible hyperbranched scaffolds, one could extend the repertoire of similar catalysts to other chemical evolution models. With some modifications, the hyperbranched scaffolds could be adjusted to study the possible chemical evolution of FeS or MoS cluster-bearing proteins, e.g., ferredoxins, hydrogenases, and nitrogenases [67]. The concept of hyperbranched scaffolds could also be broadly applicable to models of chemical evolution. In these models, cofactors other than metal sulfides, i.e., small molecules and cations, could be used. Furthermore, in lieu of hyperbranched polyimides and polyesters, other polymers could be utilized, i.e., hyperbranched polyamidoamines [68] and polypeptides [69]. 

The structure of cofactors scaffolded by globular polymers, ostensibly similar to contemporary enzymes, is intriguing in the context of chemical evolution. When considering enzyme-like prebiotic catalysts, the abundance of particular small-molecule cofactors, inorganic clusters, and cations are conceivable; however, functional high proteins and RNA molecules are unlikely to have been present at the early stages of chemical evolution. Several studies have tackled the prebiotic formation of peptide [70,71] and phosphodiester bonds [16,72]. These studies, however, so far have not addressed the mechanisms controlling the primary structure of a biopolymer that is responsible for folding and function. The function of the hyperbranched scaffolds does not depend on a precise monomer sequence and tolerates isomeric heterogeneity.

In summary, we have presented a straightforward process of a formation of stabilized polymer-supported nanoparticles. The structure of these materials, a catalytic agent scaffolded by globular hyperbranched polymers, is superficially reminiscent of the enzymatic structure and therefore is a compelling model for the study of the chemical evolution of enzymes. The hyperbranched polymer scaffold could have been the early primitive augmenting scaffold to be replaced with more sophisticated ones throughout chemical evolution. This primitive scaffold furthermore provides a means to study the aspects of small particle catalysis in prebiotic chemistry. To this end, further detailed characterizations of the hyperbranched polymer–nanoparticle aggregates are currently underway in our laboratory. Although herein we have only explored the photocatalytic ZnS NC complex relevant to the “Zinc world” hypothesis of the origin of life, the model can be easily extended to other protoenzymatic systems.

## Figures and Tables

**Table 1:** Loading capacity of Co^2+^ of hyperbranched polyesters, polyester-based dendrimers, and HyPEI.

Polymer	Co^2+^ Loading Capacity (μmol Co^2+^/g Polymer)
Hyperbranched Polyethyleneimine Mw = 800	736
COOH-terminated oligo-2,2-bis(hydroxymethyl)propionic acid dendrimer, trimethylol propane core, generation 1, C_45_H_62_O_30_ (bis-MPA-COOH)	4.5
OH-terminated oligo- 2,2-bis(hydroxymethyl)propionic acid dendrimer, trimethylol propane core, generation 1, C_21_H_38_O_12_ (bis-MPA-OH)	1.6
Citric acid–glycerol polymer, prepared neat	Non-detectable
Citric acid–glycerol, prepared in the presence of CoCl_2_	542

Bis-MPA: oligo-2,2-bis(hydroxymethyl)propionic acid.

## Supplementary Materials

The following are available online at https://www.mdpi.com/2075-1729/10/8/150/s1: Figure S1: Additional TEM views of HyPEI-ZnS NC materials, Figure S2: MALDI mass spectrum of the ZnCl_2_-bearing glycerol citrate polyester, Figure S3: Time-lapse measurement of photodegradation of eosin B (5.0 × 10^−5^M, 30 mL) under additional conditions, Figure S4: Visual progression of the eosin B degradation assay catalyzed by unsupported ZnS.
