# Supplementary material for: Protoenzymes: The Case of Hyperbranched Polymer-Scaffolded ZnS Nanocrystals
Source: Life (Basel). 2020 Aug 13;10(8):150. doi: 10.3390/life10080150 (PMC7460482; doi:10.3390/life10080150)
Supplement: Supplementary file 1 [file life-10-00150-s001.pdf]

Supplementary Materials

Protoenzymes: The Case of Hyperbranched Polymer-Scaffolded ZnS Nanocrystals

Irena Mamajanov,[1,*] Melina Caudan,[1] Tony Z. Jia[1,2]

[1] Earth Life Science Institute, Tokyo Institute of Technology, Meguro, Tokyo 152-8550, Japan.
[2] Blue Marble Institute for Science, 1001 4th Ave, Suite 3201, Seattle, WA 98154, USA.
* Correspondence: irena.mamajanov@elsi.jp

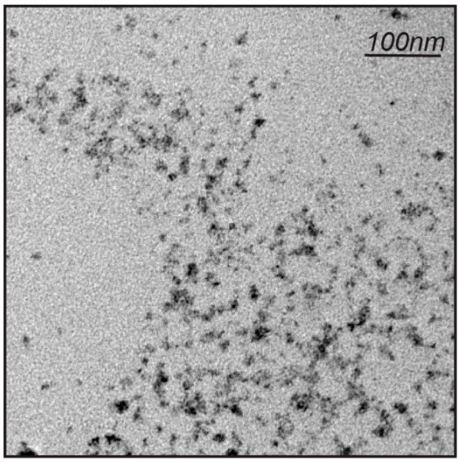

(**a**)

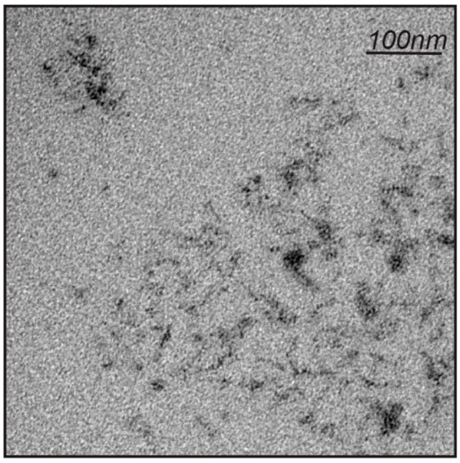

(**b**)

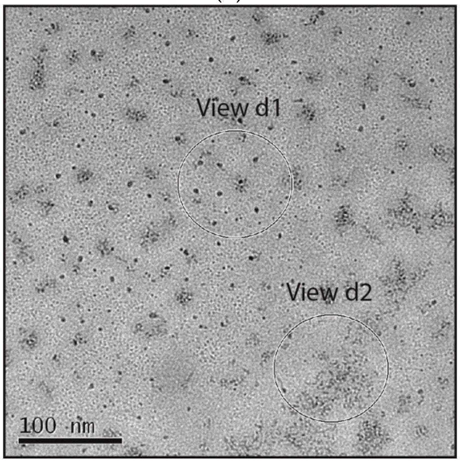

(c)

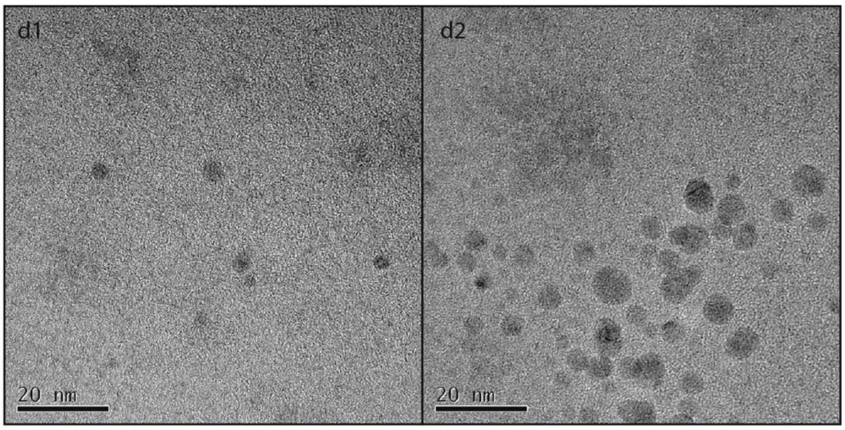

(d)

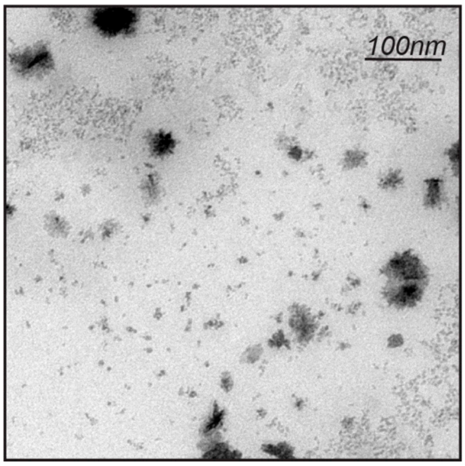

(e)

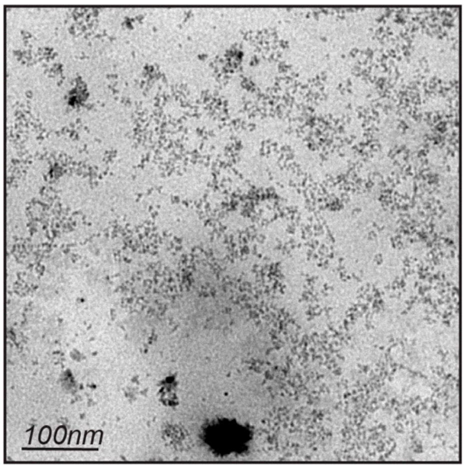

(f)

Figure S1. TEM images of HyPEI-supported ZnS nanocrystals. **(a,b)** Low magnification images of a freshly prepared sample as seen by Hitachi H7650 Zero A TEM instrument; **(c)** Low magnification FE-TEM micrograph of a freshly prepared sample as seen by a JEOL JEM-2010F FE-TEM instrument; **(d1,d2)** High magnification FE-TEM micrographs of the locations specified in **(c)** as seen by a JEOL JEM-2010F FE-TEM instrument**; (e,f)** Low magnification images of a sample aged for 14 days as seen by a Hitachi H7650 Zero A TEM instrument.

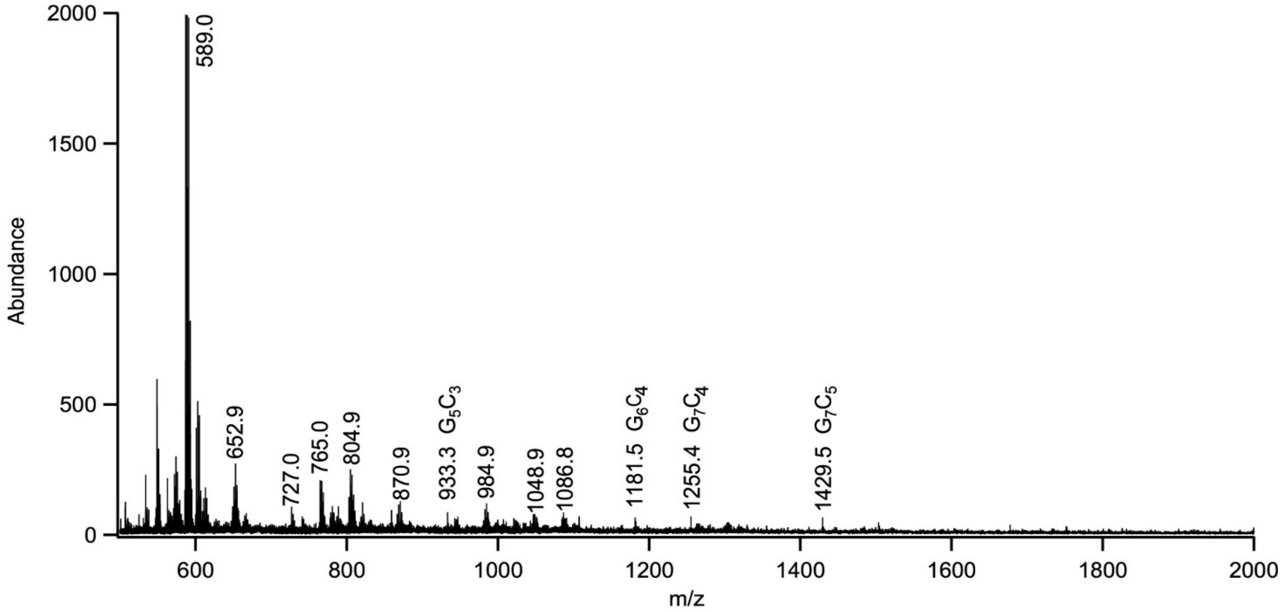

Figure S2. MALDI mass spectrum of the $ZnCl_2$-bearing glycerol citrate polyester. The mass spec is indicative of a heterogeneous mixture of polymeric species. The assigned peaks indicate the masses consistent with molecular formulae of x glycerol (G) units and y citrate units (C). The assignments were possible in the case of sodiated signals; zinc complexes were difficult to assign unequivocally due to the complexity of the zinc isotopic pattern.

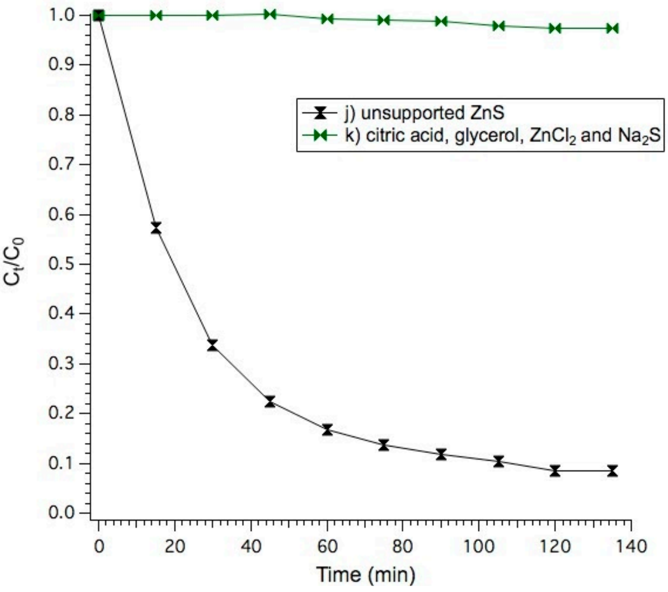

Figure S3. Time-lapse measurement of photodegradation of eosin B ($5.0x10^{-5}$M, 30mL) under different conditions: j) with unsupported ZnS particles, under UV, k) with *unreacted* citric acid, glycerol, $ZnCl_2$, Na_2S, under UV.

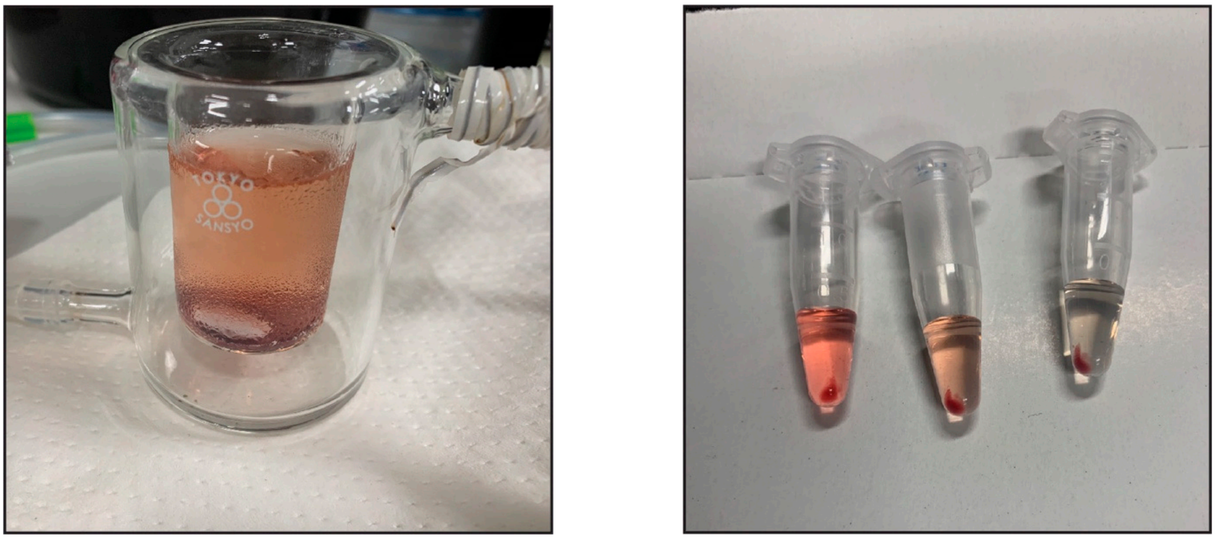

(**a**) (**b**)

Figure S4. Progression of the eosin B degradation assay catalyzed by unsupported ZnS. **(a)** Photograph of the reaction vessel showing colored precipitate at the end of the reaction. **(b)** Photograph of the centrifuged aliquots taken over the course of the measurement (left to right indicates increasing time) featuring the colored precipitate.
